# Cellulitis and transient bacteremia by *Capnocytophaga canis* after a cat scratch in an immunocompetent patient

**DOI:** 10.1099/acmi.0.000352

**Published:** 2022-05-18

**Authors:** Domingo Fernández Vecilla, Estíbaliz Ugalde Zarraga, Mikel Joseba Urrutikoechea-Gutiérrez, Francesco Renzi, José Luis Díaz de Tuesta del Arco

**Affiliations:** ^1^​ Basurto University Hospital, 18 Avenida Montevideo, 48013, Bilbao (Vizcaya), Spain; ^2^​ University of Namur, Department of Biology, Namur Research Institute for Life Sciences (NARILIS), Research Unit in Biology of Microorganisms (URBM), 61 Rue de Bruxelles, 5000 Namur, Belgium

**Keywords:** *Capnocytophaga*, *Cellulitis*, bacteremia, Capsular polysaccharide, 16S rRNA sequencing

## Abstract

*
Capnocytophaga canis* is still a rare cause of infection. We present a case of an immunocompetent patient admited in the hospital with functional impotence, pain and erythema in his left leg after suffering two scratches from his cat 48 h ago. After obtaining blood and wound cultures, broad-spectrum antimicrobial therapy with intravenous amoxicillin clavulanate was initiated. After 1 day and with a clear improvement of the symptoms the patient was discharged from the hospital with cellulitis and transient bacteremia as diagnosis and completing 1 week of antimicrobial therapy orally. After 80 and 92 h of incubation, both anaerobic flasks were positive. In the Gram-stain Gram-negative rod-shaped bacteria could be observed. Despite subculturing in brucella blood agar, tripticase soy agar with 5 % of sheep blood and chocolate agar, in both anaerobic and microaerophilic conditions, the strain could not be recovered. However, these Gram-negative rods could be identified as *C. canis* by 16S rRNA sequencing, Capsular typing was performed to study the strain, but none of the studied capsule-types tested positive. *C. canis* is still a rare cause of human infection, but it must be considered in the differential diagnosis of infections related to bites, scratches and licks from dogs or cats.

## Abbreviationsh

PCR, polymerase chain reaction; rRNA, Ribosomal RNA.

## Case report

A 72-year-old patient presents sudden pain, functional impotence and swelling in his left leg. The patient refers to a cat scratch in the left ankle 3 days before. As relevant medical history, he underwent a total thyroidectomy and bilateral lymph node dissection (VI and VII levels) 15 months before this episode due to a non-angioinvasive follicular variant papillary thyroid carcinoma (pT3a pN0), adjuvated with 3 months of radioactive iodine.

At the examination, the patient had oedema in the left dorsal foot, two little dry wounds (2×5 mm) with no fluctuation and an erythematous area that extended to the knee. He also presented a low-grade fever (37.4 °C). Blood analysis revealed a moderate leucocytosis (13000 /µl [4500–11000 µl]) with neutrophilia (10790 /µl [2000–5000 µl]), as well as a high C-reactive protein (73.29 mg l^−1^ [0–5 mg l^−1^]). The physicians obtained blood and wound cultures from the patient and then the patient was admitted to the hospital for receiving intravenous broad-spectrum antimicrobial therapy with amoxicillin/clavulanic acid 875 mg/125 mg every 8 h intravenously. An x-ray test was performed in order to rule out periosteal involvement. After a day of treatment, the pain completely disappeared and the oedematous was restricted to the dorsal and inner malleolar area of the foot. Therefore, the patient was discharged with antibiotic therapy consisting of 875/125 mg of amoxicillin/clavulanic acid each 8 h orally, fulfilling 7 more days.

**Fig. 1.**
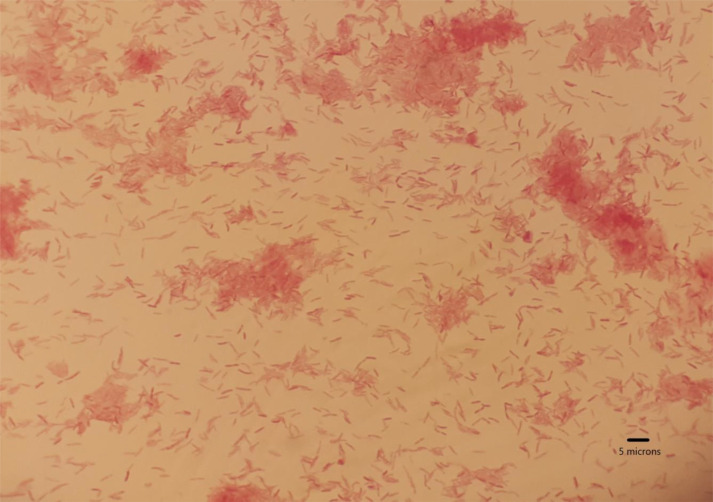
Gram-negative rod-shaped bacteria could be seen in both anaerobic bottles of the blood culture after 3 days on incubation (scale bar 10×100).

Three days and 8 h after the blood culture was obtained, the blood culture system (BD BACTEC) yielded a positive result in one of the anaerobic bottles (BD BACTEC Lytic/10 Anaerobic/F Culture Vials), whereas the other anaerobic bottle took 12 h more to result positive. In the Gram-staining of both bottles a Gram-negative rod-shaped bacteria could be seen (Fig. 1). The blood culture was inoculated in brucella blood agar with Hemin and Vitamin K1 (Becton Dickinson, Franklin Lakes, NJ, USA), tripticase soy agar with 5 % of sheep blood (Becton Dickinson, Franklin Lakes, NJ, USA) and chocolate agar (Becton Dickinson, Franklin Lakes, NJ, USA) in both anaerobic and microaerophilic conditions. There was no growth after 10 days and the blood culture was inoculated again in the same agar plates and conditions, but the strain could not be finally recovered. There were no findings in the wound cultures after 7–10 days on incubation in microaerophilic and anaerobic conditions either. Meanwhile, 500 µl of the positive blood culture was extracted on the MagNA Pure Compact System (Roche, Spain) and the 16S rRNA was sequenced as described by Oldham *et al*. [1]. The obtained sequenced was analysed with the Basic Local Alignment Search Tool (blast) and identified as *C. canis* [2]

**Fig. 2.**
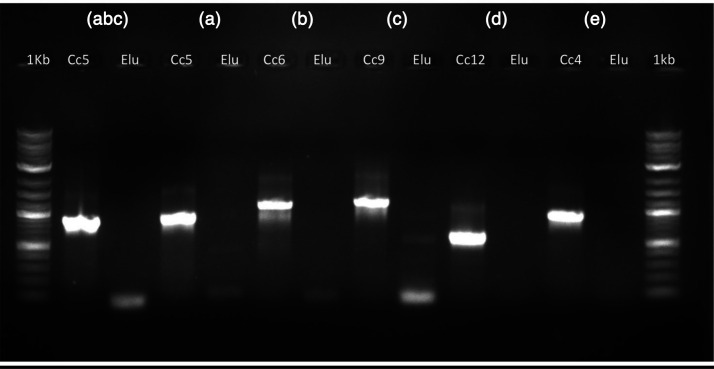
Capsular typing by PCR performed on the DNA eluate obtained from the patient blood culture. Capsular serovars (a)–(e) detection by PCR was performed as described by Hess *et al.* Elu: DNA eluate; Cc5: *C. canimorsus* strain five serovar (a); Cc6: *C. canimorsus* strain six serovar (b); Cc9: *C. canimorsus* strain nine serovar (c); Cc12: *C. canimorsus* strain 12 serovar (d); Cc4: *C. canimorsus* strain four serovar (e). The DNA eluate is negative for all serovar PCR.

In order to confirm the species identification and determine if the studied *C. canis* belonged to a known capsular-serovar, the DNA eluate was sent to the ‘*Research Unit in Biology of Microorganisms*’ of the University of Namur, Belgium. *C. canis* identification was confirmed by 16S rRNA sequencing. The capsular serovar was analysed by the PCR-typing method developed by Hess *et al.* but the results were negative for all tested serovars (A, B, C, D and E) (Fig. 2) [3], therefore, the strain might belong to another capsular serovar. However, a false negative result due to a low DNA concentration in the eluate cannot be ruled out.

## Discussion

*C. canis* is a Gram-negative rod-shaped bacteria that may grow in microaerophilic or anaerobic conditions. It belongs to the family *Flavobacteriaceae* and can be found as commensal oropharyngeal flora of healthy dogs and cats. In 2015, Renzi *et al.* performed an analysis of 105 strains previously identified as *Capnocytophaga canimorsus *, which had been isolated from different sources and countries (19 strains were isolated from human infections and 86 from dogs). 16S rRNA sequencing allowed them to build a phylogenetic tree, which revealed two main groups, with one of the groups withholding all the clinical isolates. This suggested the presence of different species that was indeed confirmed by the whole-genome sequencing and comparison of ten strains. This analysis allowed the identification of a novel *Capnocytophaga* species, named *C. canis* that appeared to be taxonomically closer to *Capnocytophaga cynodegmi* than to *C. canimorsus* [4].

All the 19 strains isolated from human infections belonged to the *C. canimorsu* s species and the *C. canis* were all isolated from dogs suggesting that this novel species could be less pathogenic than *C. canimorsus* for humans. Indeed, since its recent description*, C. canis* has rarely been related to human infections [5–7], this could be explained by the absence of some important virulence factors from *C. canimorsus* [4, 8], such as the cytochrome-oxidase activity, the ability to acquire iron from transferrin or the capacity to proliferate in human serum [8, 9].

Besides, a capsular polysaccharide (CPS) has recently been described in *C. canimorsus* as well as the presence of several capsular serovars in the species with three of them (A, B and C) being the most virulent for humans [10, 11]. In addition, Renzi *et al.* have shown that both *C. canis* and *C. cynodegmi* may also be enveloped by a capsule, showing that this feature is not restricted to the *C. canimorsus* species [8]. In fact, they reported that one strain isolated from a human infection was caused by a capsular serovar B *C. canis *. Whereas among five *C. canis* strains isolated from the oropharyngeal flora of healthy dogs, four were serovar A and one serovar F, showing that several *C. canimorsus* capsular serovars are also present in the closely related *C. canis* species. However, more studies are needed to fully understand the role of the capsular serovars on the pathogenicity of *C. canis* nee.

The latest reports have described some human infections by *C. canis *, with four documented cases of septic shock (being one of them fatal) after cat scratches and bites or contact with a dog [4–6, 12]. In a report of 2018, in which three of the total cases were collected, the strains were preliminarly assigned to *C. canimorsus* through 16S rRNA sequencing with a confidence under 90 % [7]. Nevertheless, with the discovery of the *C. canis* species, these strains were recovered for repeating the 16S rRNA sequencing and finally identified as *C. canis* with a high confidence (99.5–99.7% pairwise identities among the three clinical strains and other *C. canis* genomes) [6]. Interestingly, all the strains of the documented cases presented a positive oxidase reaction, unlike the strains isolated from healthy dogs and cats up to now [4, 5, 7]. This suggests that the *C. canis* strains with oxidase activity could be more virulent for humans than the strains lacking it. In fact, *C. canimorsus, C. cynodegmi* and the *C. canis* strains related to human infections seem to share some characteristics like the oxidase and catalase activity. In our case, this type of biochemical characteristic was not possible to study since the strain could not be recovered from culture.

In relation to the risk factors in the patients from the cases described above, at least three of them presented risk factors that had already been related to *C. canimorsus* infections**,** as chronic and heavy alcohol consumption or splenectomy (one patient presented both) [13–16]. *C. canimorsus* infections have also been reported in immunocompetent patients [17–19]. However, in the case of *C. canis* this is to our knowledge the first reported case. The patient of our case presented a medical history of papillary thyroid carcinoma but he actually did not present risk factors for being immunosuppressed.

*C. canis* remains a rare cause of human infection, but it must be taken into account as other possible causes of infections related to bites, scratches and licks from dogs or cats. Like the other virulent species of *Capnocytophaga *, *C. canimorsus* and *C. cynodegmi, C. canis* can cause fatal outcomes in both immunocompetent and immunosuppressed patients and consequently it is important to alert about the public health risk of this recently described species, in which virulence factors and prevalence in dogs and cats must be better studied.
